# Facilitating Access to Emotions: Neural Signature of EMDR Stimulation

**DOI:** 10.1371/journal.pone.0106350

**Published:** 2014-08-28

**Authors:** Deborah Herkt, Visal Tumani, Georg Grön, Thomas Kammer, Arne Hofmann, Birgit Abler

**Affiliations:** 1 Department of Psychiatry, Ulm University, Ulm, Germany; 2 EMDR-Institut Deutschland, Bergisch Gladbach, Germany; UNC Chapel Hill, United States of America

## Abstract

**Background:**

Eye Movement Desensitisation and Reprocessing (EMDR) is a method in psychotherapy effective in treating symptoms of posttraumatic stress disorder. The client attends to alternating bilateral visual, auditory or sensory stimulation while confronted with emotionally disturbing material. It is thought that the bilateral stimulation as a specific element of EMDR facilitates accessing and processing of negative material while presumably creating new associative links. We hypothesized that the putatively facilitated access should be reflected in increased activation of the amygdala upon bilateral EMDR stimulation even in healthy subjects.

**Methods:**

We investigated 22 healthy female university students (mean 23.5 years) with fMRI. Subjects were scanned while confronted with blocks of disgusting and neutral picture stimuli. One third of the blocks was presented without any additional stimulation, one third with bilateral simultaneous auditory stimulation, and one third with bilateral alternating auditory stimulation as used in EMDR.

**Results:**

Contrasting disgusting vs. neutral picture stimuli confirmed the expected robust effect of amygdala activation for all auditory stimulation conditions. The interaction analysis with the type of auditory stimulation revealed a specific increase in activation of the right amygdala for the bilateral alternating auditory stimulation. Activation of the left dorsolateral prefrontal cortex showed the opposite effect with decreased activation.

**Conclusions:**

We demonstrate first time evidence for a putative neurobiological basis of the bilateral alternating stimulation as used in the EMDR method. The increase in limbic processing along with decreased frontal activation is in line with theoretical models of how bilateral alternating stimulation could help with therapeutic reintegration of information, and present findings may pave the way for future research on EMDR in the context of posttraumatic stress disorder.

## Introduction

Eye Movement Desensitisation and Reprocessing (EMDR) is a method in psychotherapy for which meta-analyses have reliably demonstrated effectiveness in treating symptoms of posttraumatic stress disorder, PTSD [1], [2]. In EMDR, the client attends to an alternating bilateral visual, auditory or sensory stimulation while confronted with emotionally disturbing material related to the traumatic episode that promoted the disorder [3], [4]. Although the contribution of eye movements or other forms of bilateral stimulation is discussed controversially [5], a recent meta-analysis of 15 clinical and 11 laboratory trials on the additive effect of bilateral stimulation via eye movements came to the conclusion that processes involved in EMDR differ from other therapies based on exposure alone [6]. Eye movements guided by a visual stimulus alternating from side to side are the most common form of stimulation with the best evidence for effectiveness, but other forms like alternating tones or finger tapping are regularly used in clinical settings as feasible alternatives although their effectiveness is less clear [7], [8]. As EMDR uses highly standardized protocols [3], the method seems easily adaptable also to experimental approaches. In line with clinical findings of decreased vividness and arousal related to trauma-associated stimuli after EMDR, neuroimaging studies reported decreased activation of limbic areas and increased activation of prefrontal brain regions related to cognitive control after completion of successful treatments [9], [10]. However, these post-treatment studies only allowed for speculations about what happens during EMDR stimulation itself and how the effect was promoted. Neurophysiological measures [11] and electroencephalography [12], [13] have helped to uncover some potential mechanisms of action but these studies were mainly conducted in patients such that illness related neural activity may bias the specific neural signature of bilateral stimulation of its own. Therefore, we set out to investigate the neurobiological correlates of bilateral stimulation as used in EMDR under laboratory conditions in a group of healthy subjects to further add to the uncovering of basic principles of action of this specific treatment intervention.

Theoretical models of how and why posttraumatic stress disorders develop in some but not all victims of a traumatic event, suggest that traumatizing experiences remain non-integrated in a dissociated form in symptomatic subjects [14], [15], [16]. This has been concluded to result in typical symptoms of PTSD with often incomplete or less coherent memories of the traumatic event that recur involuntarily in the form of daytime flashbacks, panic attacks or nightmares that cannot be actively controlled. Repetitive exposition to these memories with consecutive habituation have been suggested as plausible mechanisms of action of common traumatherapy methods mainly in the framework of cognitive behavioural therapies [17]. Although EMDR involves confrontation with traumatic memories, periods of trauma confrontation are rather short and interrupted, most likely not permitting habituation. Furthermore, confrontation time in EMDR is much shorter than in other habituation based approaches [18], [19]. F. Shapiro, who developed the method, suggested that EMDR stimulation may facilitate the access and processing of the negative material related to traumatic episodes, allowing for the formation of new associative links, promoting the reintegration of dissociated memories [3]. Two explanations for the effect of bilateral alternating stimulation were suggested: (1) the stimulation could boost the processing of any emotionally laden material in general or (2) could have a specific effect just on the disintegrated information related to a traumatic episode. There are, however, observations in favour of the idea that the bilateral alternating stimulation could enhance affect and emotional processing in general. For example, bilateral alternating stimulation can also be used to enhance positive emotion processing [20], [21].

Assuming such a general effect on emotional processing, we hypothesized that bilateral alternating stimulation should associate with neural consequences that may even occur in healthy subjects and not only when emotional states are induced by traumatic memories but also when they are related to acute confrontation with highly affective stimuli. We furthermore hypothesized that if the bilateral alternating stimulation by itself has a neurobiological effect as has already been shown for techniques used in psychotherapy such as cognitive reappraisal [22] these neurobiological correlates should be likewise detectable with neuroimaging methods. Although the efficacy of auditory stimulation is less clear than that of eye movements [23], we opted on investigating the effects of bilateral alternating tones. With tones presented via headphones in the MR scanner, a non-compliance of the subject investigated to the task as could occur by not performing eye movements as instructed can be ruled out. Additionally, auditory stimulation permits the construction of a control condition with tones concurrently presented to both ears at the same frequency as the alternating tones. If bilateral alternating stimulation indeed has a specific effect other than mere distraction, concurrent bilateral stimulation should relate to different or at least weaker effects compared to alternating stimulation.

Regarding candidate brain regions for such an effect, it has been suggested that the amygdala may have a core role in the mechanism of action associated with bilateral alternating stimulation since the stimulation may facilitate impaired processing of negative emotional material similar to what has been shown for low-frequency tetanic stimulation in animal research [24], [25]. In line with this, an EEG study found indications of hyperactivation of limbic cortices upon bilateral stimulation in patients with PTSD [13]. Accordingly, we hypothesized that the putatively facilitated processing of negative emotional stimuli in healthy subjects should be reflected in an increased activation of the amygdala upon bilateral stimulation. Furthermore, a previous study using SPECT has demonstrated an effect of EMDR [9] where facilitated access to emotional processing was related to decreased activation in the right precentral frontal lobe. Medial and lateral prefrontal regions have previously been linked to top-down control processes potentially controlling amygdala activation in PTSD [26]. In line with this, decreased limbic but increased activation within the posterior dorsolateral prefrontal cortex (Brodman areas 6 and 44) as an emotion regulation area was linked to more dissociative symptoms [27]. Therefore, in case of increased amygdala activation associated with bilateral alternating auditory stimulation, decreased activation of dorsolateral prefrontal brain regions was hypothesized as an accompanying observation.

## Methods

### Subjects

We investigated 22 healthy female subjects between 18 and 31 years of age (mean = 23.5, SD = 2.52) without any history of psychiatric or neurological illness. All but one subject were right handed. Two more subjects had been enrolled, but were excluded from further analysis as they did not complete the experiment because of anxiety and tiredness in the scanner. To minimize effects of the female hormonal cycle, all subjects were scanned in the follicular phase between the first to sixth day of the menstrual cycle, or under hormonal contraception. Subjects were graduate, postgraduate or nursing students at the University of Ulm, and gave written informed consent before inclusion to the study. This study was conducted in accordance with the Declaration of Helsinki, under the terms of local legislation and was formally approved by the ethics committee of the University of Ulm.

Current or lifetime Axis I disorder was excluded by screening all subjects with a Structured Clinical Interview for Diagnosis – Axis I (SCID-I). Furthermore, participants had normal scores in questionnaires screening for depression and anxiety symptoms, i.e. the German version of the Center for Epidemiologic Studies Depression Scale(CES-D, [28]): “Allgemeine Depressionsskala”, ADS [29] with mean ADS scores of 5.59 (SD = 3.56) and the State Trait Anxiety Inventory for Adults (STAI, German Version, [30]) with mean STAI-S scores of 12.18 (SD = 5.45) and mean STAI-T scores of 10.36 (SD = 5.23). Individual preferences for the emotion regulation strategies ’reappraisal’ and ’suppression’ were assessed using the emotion regulation questionnaire [31] in its German version [32] with mean scores of 5.07 (SD = 0.71) for reappraisal and mean scores of 3.14 (SD = 0.79) for suppression.

### Task and stimuli

Subjects were presented with 30 picture stimuli selected from the International Affective System (IAPS) with disgusting or neutral content. We used negative pictures only of disgusting valence as those are not primarily supposed to induce fear. They were rated clearly negative in a pilot behavioral study in 25 subjects and were thus suited to induce a strong negative emotional stimulation and were demonstrated to reliably activate brain regions related to emotion processing in previous studies [33], [34], [35]. Pictures were presented in blocks of 3 with the same valence for 10 seconds each, resulting in a block length of 30 seconds. Blocks were separated by an interval of 30 seconds. 15 blocks of neutral and 15 blocks of disgusting pictures were presented. Each picture was presented 3 times throughout the experiment. Blocks of each valence appeared in randomized order. During the presentation of 10 of the disgusting and 10 of the neutral blocks, subjects were presented with concomitant auditory stimuli via headphones: The tones used for auditory stimulation were either presented simultaneously on both sides at a frequency of 1.3 Hz or alternating from left to right similar to bilateral stimulation as used for EMDR. 5 of the disgusting and 5 of the neutral blocks were presented without any auditory stimulation. The auditory stimulation was inspired by the tones produced by the AudioScan 2000 (NeuroTek Corporation), a commercially available device for auditory or tactile stimulation as used in EMDR. It is a plucked guitar tone with a fundamental frequency of 196 Hz at a duration of 250 ms repeated every 750 ms. The set of pictures presented in different versions with alternating stimulation in one third of the subjects was presented with simultaneous stimulation to another third and no auditory stimulation to the rest, i.e. over the whole group, each picture was equally presented with each type of stimulation.

After scanning, subjects were asked to rate the valence of each picture stimulus at a scale from 1 (very negative) to 9 (neutral) (“how negative or neutral was the picture”) and how they were emotionally affected by the pictures with 1 (not affected) to 9 (very affected) (“How much does the picture affect you emotionally”).

### Image acquisition methods, preprocessing and analysis

All magnetic imaging (MRI) data were obtained with a 3-Tesla Magnetom Allegra (Siemens, Erlangen, Germany) MRI Systems equipped with a head volume coil at the Department of Psychiatry of the University of Ulm. We obtained 905 volumes of functional images using an echo-planar pulse sequence (EPI). Each volume comprised 35 axial slices covering the whole cerebrum (TR/TE = 2000 ms/33 ms, 64×64 matrix). Slice thickness was 2.5 mm with no gap resulting in a voxel size of 3.6×3.6×2.5 mm. Visual stimuli were presented with LCD video goggles (Resonance Technologies, Northridge, CA), auditory stimuli via headphones (SereneSound, Resonance Technology Inc). Additionally, we acquired three-dimensional T1 weighted anatomical volumes (1×1×1 mm voxels) for each subject.

Image processing and statistical analysis were carried out using Statistical Parametric Mapping (SPM8, Welcome Trust Centre for Neuroimaging, London, UK). Preprocessing of the individual functional scans included realignment to correct for motion artifacts, slice timing, spatial normalization to a standard template (Montreal Neurological Institute, MNI) using the diffeomorphic anatomical registration through exponentiated lie algebra (DARTEL) toolbox implemented in the software and smoothing with an 8 mm FWHM Gaussian kernel. Intrinsic autocorrelations were accounted for by an AR(1) model, and low frequency drifts were removed via high pass filtering.

After preprocessing, first level analysis was performed on each subject estimating the variance of voxels according to the General Linear Model. We defined regressors corresponding to each of the six types of blocks presented (disgusting pictures with alternating tones, disgusting pictures with simultaneous tones, disgusting pictures without tone, neutral pictures with alternating tones, neutral pictures with simultaneous tone, neutral pictures without tone) According to their actual durations, trials were modeled as timely extended events and convolved with the hemodynamic response function. The 6 realignment parameters modeling residual motion were also included in the individual models. The contrast images for the 6 conditions were then included in a second level group analysis using a 2×3 ANOVA model with affect (disgusting, neutral) as the first factor with two levels. Auditory stimulation (simultaneously, alternating, none) was added as the second factor with 3 levels to test on significant interaction effects of auditory stimulation on the contrast of disgusting vs. neutral visual stimuli. To ensure that all specific interaction effects were confined to emotional stimulation, an explicit mask from the contrast of disgusting minus neutral picture stimulation over all auditory conditions was computed. The mask was thresholded at p<0.001 and only clusters that survived FDR corrections for multiple comparisons were included in further analyses. Significant interaction effects were inferred within this mask. Clusters are reported that survived false-discovery rate (FDR) corrections for multiple comparisons with the nominal level of significance at the voxel-level set at p<0.001. To exert further control on the risk of type-I errors we applied small volume corrections for multiple comparisons for the a priori defined brain regions.

## Results

### Questionnaires

Regarding subjective ratings of unpleasantness and emotional affectedness, values for disgusting pictures in each participant were computed relative to the ratings of the neutral pictures in this same participant. On average, relative unpleasantness was rated slightly higher for alternating stimulation with 5.14 (SE = 0.32) than for simultaneous stimulation with 4.96 (SE = 0.27), and similar to the condition without any auditory stimulation with 5.12 (SE = 0.23). An ANOVA on individual difference scores with factor stimulation revealed no significant effect (F(2,20) = 0.26; p = 0.78).

**Figure 1:**
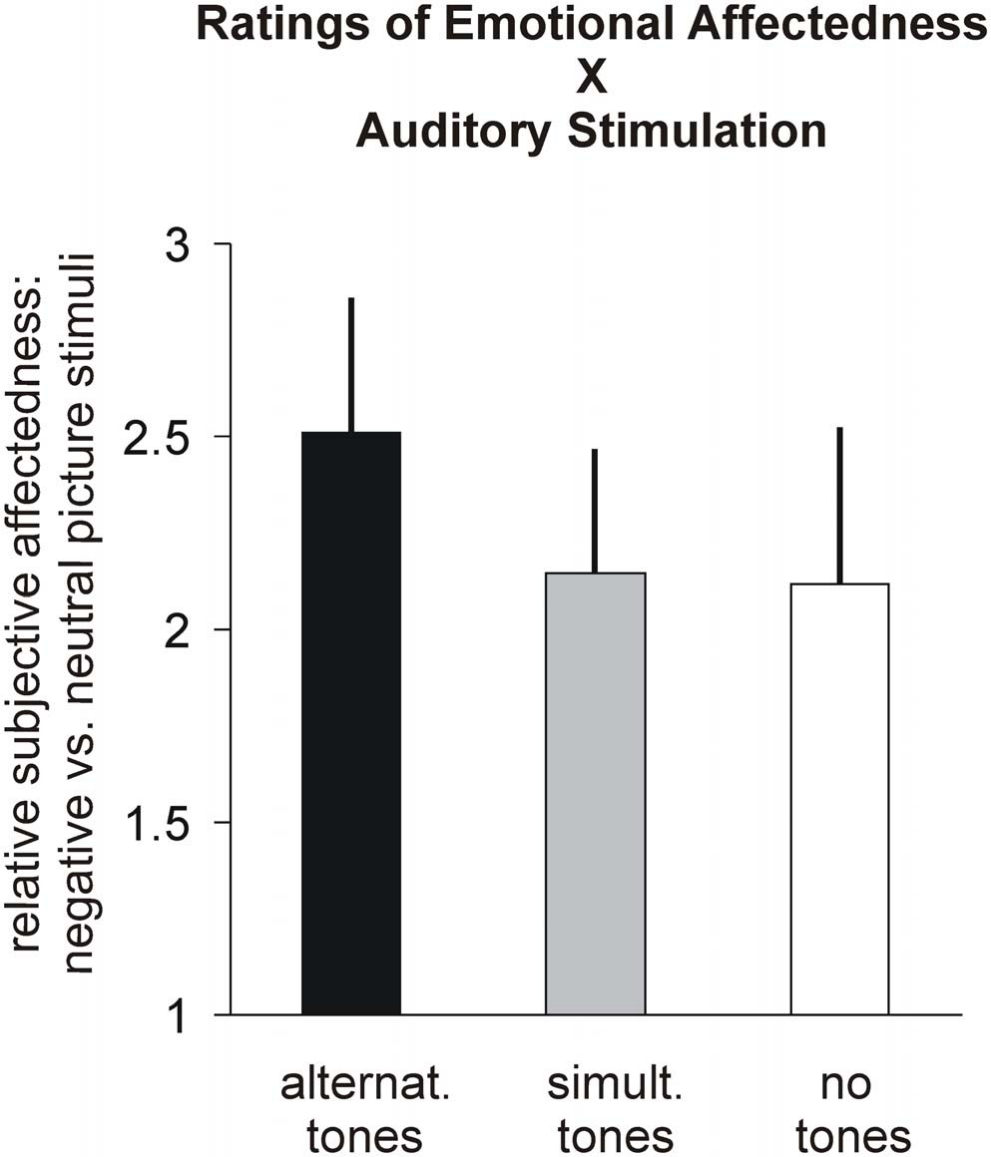
Subjective affectedness by emotional pictures. Relative ratings of how much subjects felt emotionally affected by the pictures depending on the type of auditory stimulation. Subjects were asked for their statements after scanning using a paper and pencil questionnaire showing the same pictures as in the scanner in a randomized order (the order was different from that in the scanner) and without a link to the auditory stimulation used before. Depicted are the mean differences of individual ratings of emotional vs. neutral pictures and standard errors. No significant differences were found.

Relative emotional affectedness was greatest for alternating stimulation as compared to both simultaneous stimulation or no stimulation (Figure 1) although an ANOVA with factor ‘stimulation’ revealed no significant differences between stimulation conditions (F(2,20) = 0.49; p = 0.61).

### fMRI data

Contrasting disgusting with neutral pictures under the three different auditory conditions separately revealed activation in brain regions previously related to the processing of emotions in studies with the same stimuli [33], [36]. In particular a conjunction analysis confirmed that the bilateral amygdala, hippocampus/parahippocampus, thalamus, ventrolateral and dorsolateral frontal cortices, medial prefrontal cortices, bilateral inferior and superior parietal and occipital cortices were more active upon the viewing of disgusting as compared to neutral pictures in all three auditory stimulation conditions. All cortical activation clusters survived FDR corrections (p<0.05) for multiple while small volume corrections (FWE, p<0.05) were successfully applied for subcortical regions i.e. the amygdala, hippocampus and thalamus.

**Figure 2:**
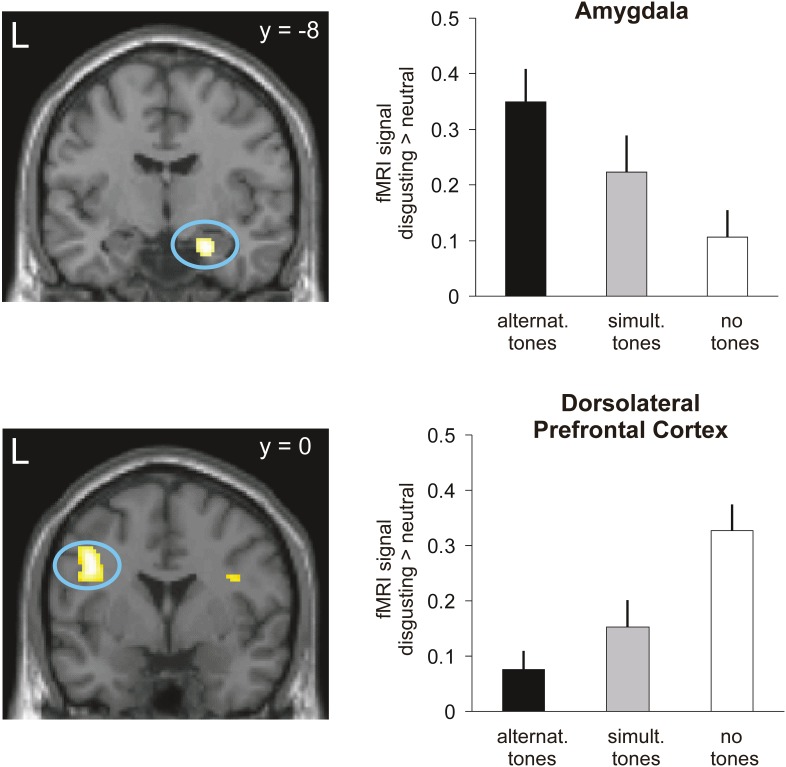
fMRI results: interaction of emotional picture presentation with type of auditory stimulation. Results of the interaction analysis of condition (visual disgusting vs. neutral stimulation) and auditory stimulation (alternating/simultaneous/no tones). Upper part – increased amygdala activation under alternating tones. Lower part – decreased dorsolateral prefrontal activation under alternating tones Plots show the mean signal of all significant voxels in the respective region and standard errors. For demonstration purposes, brain activations are shown at p<0.005 uncorrected.

An ANOVA calculated over the whole brain to test on significant interaction effects of auditory stimulation, pointed towards significant effects in the right amygdala, left dorsolateral prefrontal cortices (BA6 and BA44/45), left angular gyrus (BA40) and the right fusiform gyrus. Post-hoc t-tests (p<0.001, >10 voxels per cluster) revealed that during bilateral alternating stimulation, the amygdala activation was relatively increased as compared to no auditory stimulation, while dorsolateral prefrontal cortex activation was relatively decreased as compared to both, bilateral simultaneous stimulation and no auditory stimulation (Table 1, Figure 2). Activation within the regions of interest, i. e. the amygdala and dorsolateral prefrontal cortex survived small volume corrections (FWE, p<0.05).

**Table 1:** Condition-by-auditory stimulation interaction effects.

	alternating>notones			no>alternatingtones			no>simultaneoustones		
disgusting vs. neutralpicture stimuli	Z	NV	PeakCoord.	Z	NV	PeakCoord.	Z	NV	PeakCoord.
			x/y/z			x/y/z			x/y/z
Amygdala, right	3.31	11	22/–8/–22#						
Dorsolateral prefrontalcortex, BA6, left				4.18	168	–42/0/34#	3.53	46	–44/0/38#
Dorsolateral prefrontalcortex, BA44/45, left				3.47	19	–54/12/32#	3.69	33	–54/12/32#
							3.90	41	–46/24/10#
Angular gyrus,BA40, left				3.27	14	–32/–44/36			
Fusiform gyrus							3.38	16	40/–76/4

Z: z-score; NV: number of continguously significant voxels; peak coordinates of clusters are are MNI (Montreal Neurological Institute) normalized stereotactic coordinates: –x: left from the anterior commisure (AC); –y: posterior from AC; –z: inferior from AC; BA: Brodmann area; no significant interactions for alternating>simultaneous tones, simultaneous>alternating tones, simultaneous>no tones.

Results of the whole brain analyses are reported at p<0.001 uncorrected with a threshold of >10 voxels per cluster.

# Regions of interest that survived small volume corrections for multiple comparisons (FWE, p<0.05).

## Discussion

In the present study, we demonstrate first time evidence for a putative neurobiological basis of the bilateral alternating stimulation as used in the framework of the EMDR method in healthy subjects. The increase in limbic processing with greater activation of the amygdala during the processing of negative emotional stimuli along with indicated decreased activation in dorsolateral prefrontal brain areas related to cognitive control mechanisms [27] may support theoretical models of how bilateral alternating stimulation can help with the processing of traumatic memories. Subjective ratings of how much subjects felt touched by the affective stimuli were numerically increased. This further supports the notion of enhanced emotional processing under bilateral alternating stimulation.

While the EMDR method as a whole may relate to a more ample interplay of different mechanisms of action, we demonstrate that the specific element of the method, i. e. the bilateral alternating stimulation has a distinct neurobiological effect on negative emotion processing. This observation stands against previous statements suggesting that the bilateral stimulation in EMDR may be a dispensable element of the method [37], [38] and may be suited to challenge the initial scepticisms regarding the contribution of this element [5]. Replicating previous findings of Servan-Schreiber et al. [11], our results point towards the idea that not only bilateral alternating but also bilateral concomitant stimulation at the same frequency has a similar, although weaker effect. This finding, together with findings like those by [39] and [40] who investigated effects of bilateral alternating horizontal and vertical stimulation as well as distraction by taxing working memory, was interpreted in the context of the effects on emotional processing during dual attention tasks in non-clinical populations [41], [42]. Dual attention tasks were shown to decrease the vividness of concomitant emotional aspects. Limited working memory capacity has been suggested as a reason for this [43]. This interpretation seemed feasible, as the studies investigated the after-effect of bilateral stimulation or dual tasking during the processing of traumatic events. Patients’ subjective distress was assessed after the stimulation and, as expected with EMDR, was seen to decrease. However, our results suggest that in line with theoretical models of how bilateral stimulation acts [3], [4], initially enhanced, not decreased, emotional processing may be a prerequisite for this subsequent decrease in subjective distress. Imaging experiments of dual attention tasks showed a relation of increasing working memory load with decreased activation of the amygdala [44]. In the present study, we observed an increase in activation of the amygdala as the core region processing emotional stimuli and a decrease in activation of left-lateralized prefrontal brain regions previously related to working memory processes and active cognitive control like the suppression of unwanted memories [45].

Although the emotional reaction upon triggering traumatic memories in patients with PTSD is not the same as the emotional reaction of healthy subjects to disgusting pictures, brain activation patterns in both cases overlap greatly: Brain regions linked to emotion processing in healthy subjects include the amygdala, insula, hippocampus, orbitofrontal, medial and lateral prefrontal and cingulate cortices [46] and have been related to PTSD as well [47], [48]. In particular, activation within this network was linked to PTSD symptomatology even when the negative material was not related to traumatic events [47]. For another condition with such an overlap of brain networks involved [49], [50], i.e. acute pain in healthy subjects and chronic pain in patients, highly similar effects of pain medication on activation patterns have been found [51], [52]. We therefore think that the mechanisms identified for bilateral alternating stimulation in healthy subjects might at least in part mirror effects in patients with PTSD. Actually, our findings are well in line with previous findings regarding neurobiological correlates of bilateral stimulation assessed using EEG in patients [13]. In this study, increased limbic activation was shown upon initial stimulation along with decreases upon successful processing of traumatic material after therapy.

Dissociation is a core symptom of PTSD and potentially a protective mechanism that occurs upon acute confrontation with negative emotional or traumatic material as well as upon triggering memories related to negative events that relates to a subjective dampening of affect and emotions related to the acute event or memory [53]. These protective mechanisms are frequently described to interfere with psychotherapy, particularly the highly effective confrontational techniques. Emotional engagement with the information related to the traumatic event has been described as essential for exposure-based treatments to be successful [54]. So far, neuroimaging research on posttraumatic stress disorder (PTSD) identified a pattern of opposing activations of limbic system and prefrontal cortex activation as typical for PTSD [55] and particularly the symptom of dissociation [27]. Investigations showing relatively increased activation in brain areas related to prefrontal top-down cognitive control in patients with PTSD with dissociative symptoms [56] suggest an excessive corticolimbic inhibition leading to an emotional overmodulation [26]. Accordingly, it was shown that in PTSD related to interpersonal violence, the severity of dissociative symptoms was positively correlated with increased activation in the dorsolateral prefrontal cortex and negatively to the activation of the amygdala [27]. Thus, our findings that auditory stimulation as used in EMDR decreases prefrontal and increases limbic activation may inspire further research on potential mechanisms counteracting activation patterns related to dissociation in patients. Such investigations might help to explain why the processing of negative emotional material during EMDR sessions can occur with greater speed than with other methods also using trauma confrontation but without concomitant bilateral stimulation. Findings of increased emotional processing would be in line with theoretical models about the effectiveness of bilateral stimulation [3], [4], proposing that initially enhanced, but not decreased, emotional processing is a prerequisite for subsequent decrease in subjectively experienced distress.

## Conclusion

In the present study we have investigated the effect of auditory stimulation as used in EMDR while processing stimuli of negative emotional valence. We observed a parametric effect with bilateral alternating auditory stimulation leading to the greatest increase in activation of the amygdala and the greatest decrease in activation of dorsolateral prefrontal brain areas compared to simultaneous auditory stimulation and no stimulation at all. These results in healthy control subjects may encourage investigations in patients whether exposure therapies with concomitant bilateral stimulation indeed engage a neurobiologically grounded mechanism that may be beneficial to force open dissociation as one of the primary defence strategies often interfering with therapy in PTSD.

## References

[1] Bisson J, Andrew M (2007) Psychological treatment of post-traumatic stress disorder (PTSD). Cochrane Database Syst Rev: CD003388. 10.1002/14651858.CD003388.pub2 15846661

[2] Watts BV, Schnurr PP, Mayo L, Young-Xu Y, Weeks WB, et al (2013) Meta-analysis of the efficacy of treatments for posttraumatic stress disorder. J Clin Psychiatry 74: e541–550. 23842024 10.4088/JCP.12r08225

[3] Shapiro F (1995) Eye Movement Desensitization and Reprocessing. Basic principles, protocols, and procedures. New York: Guilford Press.

[4] Shapiro F (2002) EMDR 12 years after its introduction: Past and future research. Journal of Clinical Psychology 58: 1–22. 11748594 10.1002/jclp.1126

[5] Cahill SP, Carrigan MH, Frueh BC (1999) Does EMDR work? And if so, why?: a critical review of controlled outcome and dismantling research. J Anxiety Disord 13: 5–33. 10225499 10.1016/s0887-6185(98)00039-5

[6] Lee CW, Cuijpers P (2013) A meta-analysis of the contribution of eye movments in processing emotional memories. Journal of Behavioral Therapy and Experimental Psychiatry 44: 231–239. 10.1016/j.jbtep.2012.11.001 23266601

[7] Maxfield L (2008) Considering mechanisms of action in EMDR. Journal of EMDR Practice and Research 2: 234–238.

[8] van den Hout MA, Engelhard IM, Rijkeboer MM, Koekebakker J, Hornsveld H, et al (2011) EMDR: eye movements superior to beeps in taxing working memory and reducing vividness of recollections. Behav Res Ther 49: 92–98. 21147478 10.1016/j.brat.2010.11.003

[9] Lansing K, Amen DG, Hanks C, Rudy L (2005) High-resolution brain SPECT imaging and eye movement desensitization and reprocessing in police officers with PTSD. Journal of Neuropsychiatry and Clinical Neuroscience 17: 526–532. 10.1176/jnp.17.4.526 16387993

[10] Pagani M, Högberg G, Salmaso D, Nardo D, Tärnell B, et al (2007) Effects of EMDR psychotherapy on 99mTc-HMPAO distribution in occupation-related post-traumatic stress disorder. Nuclear Medicine Communications 28: 757–765. 17728604 10.1097/MNM.0b013e3282742035

[11] Servan-Schreiber D (2006) Schooler J, Dew MA, Carter C, Bartone P (2006) Eye movement desensitization and reprocessing for posttraumatic stress disorder: a pilot blinded, randomized study of stimulation type. Psychother Psychosom 75: 290–297. 16899965 10.1159/000093950

[12] Pagani M, Di Lorenzo G, Verardo AR, Nicolais G, Monaco L, et al (2011) Pre-, intra- and post-treatment EEG imaging of EMDR-Methodology and preliminary results from a single case. Journal of EMDR Practice and Research 5: 42–56.

[13] Pagani M, Di Lorenzo G, Verardo AR, Nicolais G, Monaco L, et al (2012) Neurobiology of EMDR-EEG imaging of treatment efficacy. PloS One 7: e45753. 23049852 10.1371/journal.pone.0045753 PMC3458957

[14] Nijenhuis ER, van der Hart O (2011) Dissociation in trauma: a new definition and comparison with previous formulations. J Trauma Dissociation 12: 416–445. 21667387 10.1080/15299732.2011.570592

[15] van der Hart O, Nijenhuis ER, Steele K (2005) Dissociation: An insufficiently recognized major feature of complex posttraumatic stress disorder. J Trauma Stress 18: 413–423. 16281239 10.1002/jts.20049

[16] van der Kolk B (2000) Posttraumatic stress disorder and the nature of trauma. Dialogues Clin Neurosci 2: 7–22. 22034447 10.31887/DCNS.2000.2.1/bvdkolk PMC3181584

[17] Bradley R, Greene J, Russ E, Dutra L, Westen D (2005) A multidimensional meta-analysis of psychotherapy for PTSD. Am J Psychiatry 162: 214–227. 15677582 10.1176/appi.ajp.162.2.214

[18] Ho MSK, Lee CW (2012) Cognitive behaviour therapy versus eye movement desensitization and reprocessing for post-traumatic disorder – is it all in the homework then? Revue européenne de psychologie appliquée 62: 253–260.

[19] Van Etten ML, Taylor S (1998) Comparative Efficacy of Treatments for Post-traumatic Stress Disorder: A Meta-Analysis. Clinical Psychology and Psychotherapy 5: 126–144.

[20] Sprang G (2001) The Use of Eye Movement Desensitization and Reprocessing (EMDR) in the Treatment of Traumatic Stress and Complicated Mourning: Psychological and Behavioral Outcomes. Research on Social Work Practice 11: 300–320.

[21] Korn DL, Leeds AM (2002) Preliminary Evidence of Efficacy for EMDR Resource Development and Installation in the Stabilization Phase of Treatment of Complex Posttraumatic Stress Disorder. Journal of Clinical Psychology 58: 1465–1487. 12455016 10.1002/jclp.10099

[22] Staudinger MR, Erk S, Abler B, Walter H (2009) Cognitive reappraisal modulates expected value and prediction error encoding in the ventral striatum. Neuroimage 47: 713–721. 19442745 10.1016/j.neuroimage.2009.04.095

[23] van den Hout MA, Rijkeboer MM, Engelhard IM, Klugkist I, Hornsveld H, et al (2012) Tones inferior to eye movements in the EMDR treatment of PTSD. Behav Res Ther 50: 275–279. 22440458 10.1016/j.brat.2012.02.001

[24] Bender VA, Bender KJ, Brasier DJ, Feldman DE (2006) Two coincidence detectors for spike timing-dependent plasticity in somatosensory cortex. J Neurosci 26: 4166–4177. 16624937 10.1523/JNEUROSCI.0176-06.2006 PMC3071735

[25] Holscher C, Anwyl R, Rowan MJ (1997) Stimulation on the positive phase of hippocampal theta rhythm induces long-term potentiation that can Be depotentiated by stimulation on the negative phase in area CA1 in vivo. J Neurosci 17: 6470–6477. 9236254 10.1523/JNEUROSCI.17-16-06470.1997 PMC6568346

[26] Lanius RA, Vermetten E, Loewenstein RJ, Brand B, Schmahl C, et al (2010) Emotion modulation in PTSD: Clinical and neurobiological evidence for a dissociative subtype. Am J Psychiatry 167: 640–647. 20360318 10.1176/appi.ajp.2009.09081168 PMC3226703

[27] Moser DA, Aue T, Wang Z, Rusconi Serpa S, Favez N, et al (2013) Limbic brain responses in mothers with post-traumatic stress disorder and comorbid dissociation to video clips of their children. Stress 16: 493–502. 23777332 10.3109/10253890.2013.816280

[28] Radloff LS (1977) The CES-D scale: A self-report depression scale for research in the general population. Appl Psychol Measurement 1: 385–401.

[29] Hautzinger M, Bailer M (1993) Allgemeine Depressionsskala (ADS). Weinheim: Beltz.

[30] Laux L, Glanzmann P, Schaffner P, Spielberger CD (1981) Das Stait-Trait-Angstinventar. Weinheim, Germany: Beltz.

[31] Gross JJ, John OP (2003) Individual differences in two emotion regulation processes: implications for affect, relationships, and well-being. J Pers Soc Psychol 85: 348–362. 12916575 10.1037/0022-3514.85.2.348

[32] Abler B, Kessler H (2009) Emotion Regulation Questionnaire - Eine deutsche Fassung des ERQ von Gross & John. Diagnostica 55: 144–152.

[33] Erk S, Walter H, Abler B (2008) Age-related physiological responses to emotion anticipation and exposure. Neuroreport 19: 447–452. 18287944 10.1097/WNR.0b013e3282f5d92f

[34] Abler B, Erk S, Herwig U, Walter H (2007) Anticipation of aversive stimuli activates extended amygdala in unipolar depression. J Psychiatr Res 41: 511–522. 17010993 10.1016/j.jpsychires.2006.07.020

[35] Erk S, Abler B, Walter H (2006) Cognitive modulation of emotion anticipation. Eur J Neurosci 24: 1227–1236. 16930447 10.1111/j.1460-9568.2006.04976.x

[36] Abler B, Hofer C, Walter H, Erk S, Hoffmann H, et al (2010) Habitual emotion regulation strategies and depressive symptoms in healthy subjects predict fMRI brain activation patterns related to major depression. Psychiatry Res 183: 105–113. 20630713 10.1016/j.pscychresns.2010.05.010

[37] Lohr JM, Lilienfeld SO, Tolin DF, Herbert JD (1999) Eye Movement Desensitization and Reprocessing: an analysis of specific versus nonspecific treatment factors. J Anxiety Disord 13: 185–207. 10225508 10.1016/s0887-6185(98)00047-4

[38] Lohr JM, Tolin DF, Lilienfeld SO (1998) Efficacy of eye movement desensitization and reprocessing: implications for behavior therapy. Behav Ther 29: 123–156.

[39] Hornsveld HK, Houtveen J, Vroomen M, Aalbers I, Kapteijn I, et al (2011) Evaluating the effect of eye movements on positive memories such as those used in Resource Development and Installation. Journal of EMDR Practice and Research 5: 146–155.

[40] Van den Hout M, Engelhard IM, Smeets MAM, Horsnveld H, Hoogeveen E, et al (2010) Counting during recall: Taxing of working memory and reduced vividness and emotionality of negative memories. Applied Cognitive Psychology 24: 303–311.

[41] Andrade J, Kavanagh D, Baddeley A (1997) Eye-movements and visual imagery: a working memory approach to the treatment of post-traumatic stress disorder. Br J Clin Psychol 36 (Pt 2): 209–223. 10.1111/j.2044-8260.1997.tb01408.x 9167862

[42] Barrowcliff AL, Gray NS, Freeman TCA, Mac-Culloch MJ (2004) Eye movements reduce the vividness, emotional valence and electrodermal arousal associated with negative autobiographical memories. J Forensic Psychiatry Psychol 15: 325–345.

[43] Van den Hout M, Engelhard IM (2012) How does EMDR work? J Exp Psychopathol 3: 724–738.

[44] Clarke R, Johnstone T (2013) Prefrontal inhibition of threat processing reduces working memory interference. Front Hum Neurosci 7: 228. 23750133 10.3389/fnhum.2013.00228 PMC3667546

[45] Anderson MC, Ochsner KN, Kuhl B, Cooper J, Robertson E, et al (2004) Neural systems underlying the suppression of unwanted memories. Science 303: 232–235. 14716015 10.1126/science.1089504

[46] Phan KL, Wager T, Taylor SF, Liberzon I (2002) Functional neuroanatomy of emotion: a meta-analysis of emotion activation studies in PET and fMRI. Neuroimage 16: 331–348. 12030820 10.1006/nimg.2002.1087

[47] Etkin A, Wager TD (2007) Functional neuroimaging of anxiety: a meta-analysis of emotional processing in PTSD, social anxiety disorder, and specific phobia. Am J Psychiatry 164: 1476–1488. 17898336 10.1176/appi.ajp.2007.07030504 PMC3318959

[48] Lanius RA, Bluhm R, Lanius U, Pain C (2006) A review of neuroimaging studies in PTSD: heterogeneity of response to symptom provocation. J Psychiatr Res 40: 709–729. 16214172 10.1016/j.jpsychires.2005.07.007

[49] Bingel U, Tracey I (2008) Imaging CNS modulation of pain in humans. Physiology (Bethesda) 23: 371–380. 19074744 10.1152/physiol.00024.2008

[50] Tracey I (2008) Imaging pain. Br J Anaesth 101: 32–39. 18556697 10.1093/bja/aen102

[51] Sprenger T, Valet M, Woltmann R, Zimmer C, Freynhagen R, et al (2006) Imaging pain modulation by subanesthetic S-(+)-ketamine. Anesth Analg 103: 729–737. 16931688 10.1213/01.ane.0000231635.14872.40

[52] Wagner KJ, Sprenger T, Kochs EF, Tolle TR, Valet M, et al (2007) Imaging human cerebral pain modulation by dose-dependent opioid analgesia: a positron emission tomography activation study using remifentanil. Anesthesiology 106: 548–556. 17325514 10.1097/00000542-200703000-00020

[53] van der Kolk BA, Pelcovitz D, Roth S, Mandel FS, McFarlane A, et al (1996) Dissociation, somatization, and affect dysregulation: the complexity of adaptation of trauma. Am J Psychiatry 153: 83–93. 10.1176/ajp.153.7.83 8659645

[54] Foa EB, Kozak MJ (1986) Emotional processing of fear: exposure to corrective information. Psychol Bull 99: 20–35. 2871574

[55] Shin LM, Orr SP, Carson MA, Rauch SL, Macklin ML, et al (2004) Regional cerebral blood flow in the amygdala and medial prefrontal cortex during traumatic imagery in male and female Vietnam veterans with PTSD. Arch Gen Psychiatry 61: 168–176. 14757593 10.1001/archpsyc.61.2.168

[56] Lanius RA, Williamson PC, Boksman K, Densmore M, Gupta M, et al (2002) Brain activation during script-driven imagery induced dissociative responses in PTSD: a functional magnetic resonance imaging investigation. Biol Psychiatry 52: 305–311. 12208637 10.1016/s0006-3223(02)01367-7

